# Physical capacity, subjective health, and life satisfaction in older women: a 10-year follow-up study

**DOI:** 10.1186/s12877-021-02605-z

**Published:** 2021-11-23

**Authors:** Sarang Latif Qazi, Heli Koivumaa-Honkanen, Toni Rikkonen, Reijo Sund, Heikki Kröger, Masoud Isanejad, Joonas Sirola

**Affiliations:** 1 Kuopio Musculoskeletal Research Unit (KMRU), University of Eastern Finland, Mediteknia Building, P.O. Box 1627, 70211 Kuopio, Finland; 2 Mental Health and Wellbeing Center, Kuopio University Hospital, Kuopio, Finland; 3 Department of Orthopaedics, Traumatology and Hand Surgery, Kuopio University Hospital, Kuopio, Finland; 4 Institute of Life Course and Medical Sciences, Faculty of Health & Life Sciences, University of Liverpool, Liverpool, UK

**Keywords:** Aging, Physical capacity, Subjective wellbeing, Network analysis

## Abstract

**Background:**

Physical capacity and subjective wellbeing are important for healthy aging. Our aim was to study how objective/subjective physical capacity and subjective health relate to life satisfaction, in a 10-year follow-up of aging women.

**Methods:**

The participants (*n* = 1485, mean age 67.4 years) consisted of community-dwelling older women living in Kuopio, Finland. Grip strength and one-legged stance test time were used as objective, and self-rated mobility (SRM) as subjective physical capacity measures. Self-rated health (SRH) and SRM were assessed with one-item scales and life satisfaction with a 4-item scale. Correlation and linear regression were used to analyze these relationships and correlation network analysis to visualize them. Age and BMI were included in the analysis as adjusting factors.

**Results:**

All the study variables were significantly correlated with baseline and follow-up life satisfaction, except BMI, which was only associated with life satisfaction at follow-up. On both occasions, SRH and SRM were the two strongest correlates of life satisfaction, but their mutual correlation was still higher. In linear regression analyses, SRH was positively associated with both baseline and follow-up life satisfaction, but physical capacity measures became non-significant after including SRH and SRM in the model. In the partial correlation network analyses, SRH and SRM were the most central nodes, connecting every other variable.

**Conclusions:**

Self-reports on health, mobility, and life satisfaction are closely intertwined and provide easily accessible health information among aging women, but the impacts of objective physical capacity measures warrant further longitudinal studies in respect to subjective wellbeing among aging people.

**Supplementary Information:**

The online version contains supplementary material available at 10.1186/s12877-021-02605-z.

## Introduction

According to the World Health Organization (WHO), healthy aging is a process of developing and maintaining functional ability that enables physical, mental, and social wellbeing [1]. Subjective wellbeing (SWB) is the general term referring to the various types of subjective evaluations of one’s life, including both cognitive evaluations (such as life satisfaction) and affective feelings (such as happiness) [2]. It is also one of the main domains of good mental health [3]. In the past 20 years, SWB has become an area of active research [4, 5]. The knowledge of its relationship with physical health has increased, but less attention has been paid to its relationship with physical capacity and mobility. This also applies to life satisfaction.

Life dissatisfaction can be a risk factor for several long-term adverse health outcomes such as mortality, premature disability, and poor mental health [6–10]. On the other hand, life satisfaction can be protective against heavy alcohol use in the general population, poor treatment outcomes in psychiatric and surgical patients, and bone loss in postmenopausal women [11–14]. Life satisfaction and its correlates have been widely studied among older adults, but studies on how physical capacity or mobility measures relate specifically to life satisfaction are few, in spite of their public health importance [15].

In the present study, we used two objective (grip strength and one-legged stance test (OLST) time) and one subjective (self-rated mobility) measures for physical capacity. Grip strength is an easily assessable objective measure and is an indicator of global muscle strength, physical performance, and frailty, as well as a predictor of disability and longevity [16–19]. Thus far, a few studies on its association with life satisfaction have reported conflicting results by gender [20, 21]. OLST time is an objective measure of static, steady-state, one-legged balance and a correlate of maximal muscle strength [22]. The studies on the relationship between self-rated mobility and life satisfaction using different scales come from small study groups [15, 23–25]. Clarification of these relationships warrants further research, in which both the objective and subjective measurements of health and physical capacity are taken into account.

Self-rated health as general health perception is an individual’s synthesis of subjective and objective information about one’s health. It provides information that is not captured by using only objective measures [26]. Even though self-rated health is strongly related to SWB, they are not interchangeable. Whether one’s overall health is perceived more in terms of physical or mental health can be related to different characteristics of the study population, such as age [27].

In general, loss of physical capacity can herald a decrease in social life and a deterioration of physical and mental health in older age, whereas improvements in physical capacity could be beneficial to SWB [23]. Due to this and their effects on health care and societal costs, these relationships in old age are important*.* The aim of the present study was to investigate the relationship between physical capacity, subjective health, and life satisfaction in a large sample of aging women.

## Materials and methods

### Study cohort

**Fig. 1:**
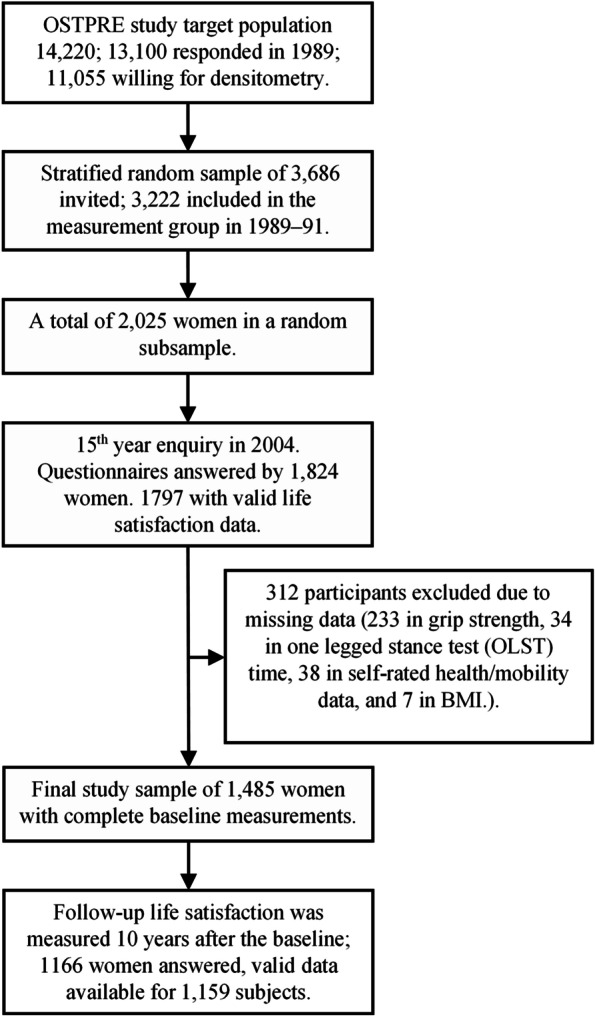
Timeline of the study. Note: OSTPRE = Kuopio Osteoporosis Risk Factor and Prevention study, OLST = One-legged stance test

This study utilized data from the Osteoporosis Risk Factor and Prevention (OSTPRE) study, which is a longitudinal study initiated in 1989. The follow-up study flow is summarized in Fig. 1. The initial target population of the OSTPRE study consisted of all the 14,220 women born between 1932 and 41 and living in the province of Kuopio, Finland. At the start of the OSTRPE study in 1989, a stratified random sample of 3686 women was selected from 11,055 respondents willing to undergo DXA, of which 3222 women underwent baseline clinical measurements. The stratified sample consisted of random (*n* = 2025) and non-random subsamples (*n* = 1197). Only the random subsample was included in the current study.

The women were subsequently followed up by means of postal health enquiries and physical measurements at five-year intervals, which provided data on the main study measures: life satisfaction, physical capacity (grip strength, single leg balance, and self-rated mobility), and others (self-rated health and body mass index) (see 2.2 – 2.4). The 15th year enquiry, which is the baseline for this study, was carried out in 2004.

Baseline OSTPRE questionnaires were answered by 1824 women. We excluded 27 women from the analyses due to incomplete data for the life satisfaction score (see scoring in 2.2) (*N* = 1797). Further, due to missing data, we excluded (stepwise) a total of 312 women (grip strength 233, one-legged stance test (OLST) time 34, self-rated health and self-rated mobility 38, and BMI 7). The final study sample consisted of 1485 women with complete baseline data. Of these, the 10-year questionnaire was returned by 1166 women, with 99.4% (1159/1166) having answered at least two life satisfaction items (see 2.2). The final follow-up sample size was 1159, and the mean follow-up time was 9.3 years.

### Life satisfaction

Life satisfaction was assessed using the four-item life satisfaction scale, which was developed by Erik Allardt in 1973 [24]. This has been used in various studies on both general and patient populations [9, 12, 25]. Its four items (each with scores 1-5) comprised the following questions:

- *Do you feel that your life at present is…?* (score)
  1. very interesting (=1), fairly interesting (=2), fairly boring (=4), very boring (=5), cannot say (=3)
  2. very happy (=1), fairly happy (=2), fairly sad (=4), very sad (=5), cannot say (=3)
  3. very easy (=1), fairly easy (=2), fairly hard (=4), very hard (=5), cannot say (=3)
- *Do you feel that at the present you are…?* (score)
  4. very lonely (=5), fairly lonely (=4), not at all lonely (=1), cannot say (=3)

If a response was missing three or four items, the life satisfaction score was recorded as missing data. In cases where a response was missing only one or two items, the missing items were recorded as 3. At the baseline, 95% (1729/1824) of the women answered all four items, whereas 98% (1797/1824) of the women responded to at least two items. The corresponding figures for the follow-up life satisfaction score were 97% (1120/1166) and 99% (1159/1166), respectively.

The life satisfaction score (range: 4–20) was calculated as the sum of all four items, with higher scores signifying lower life satisfaction. In the network analyses, the scale was inverted so that positive associations indicate better life satisfaction.

### Physical capacity

Grip strength was measured using a handgrip dynamometer (JAMAR™ handgrip dynamometer; Sammons Preston, Bolingbrook, IL). Measurements were made from the dominant hand while seated on a bench, with the forearm flexed from the elbow at a 90° angle, close to the torso. Three attempts were recorded, with 30 s of rest between each attempt. The average results were reported in kilogram force (kgF). The hand dynamometer is the standard approach to measuring grip strength in older populations [28].

OLST (i.e., single leg balance) time was recorded by asking the subjects to stand on their dominant foot for thirty seconds. Hands were placed on the hip and the weight-bearing foot was not allowed to move. The test was stopped if the subject was able to perform the test for 30 s. Two attempts were allowed, and the best attempt was recorded. The test has acceptable reliability [29].

Self-rated mobility was also measured on a Likert-type scale. Participants were asked “What is your current capability for movement?” The response alternatives were (score): fully capable of movement (=1), capable of movement but unable to run (=2), I can walk 1 km at the most (=3), I can walk 100 m at the most (=4), I can move only indoors (=5), I’m incapable of movement (=6), I’m temporarily incapable of movement (=6). They were inverted to make the results more comprehensible, meaning that higher scores indicate better self-rated mobility.

### Other measurements

Self-rated health was also measured on a Likert-type scale by asking “What is the best term to describe your current health compared to others of the same age?” Response alternatives were (score): very good (=1), good (=2), moderate (=3), not good (=4), poor (=5). They were inverted to make the results more comprehensible, meaning that higher scores indicate better self-rated health.

Weight was measured in light clothing using a digital calibrated scale (type HF 351/00; Philips, Andover, MA, USA), and height was measured using a calibrated stadiometer. Body mass index was calculated as a person’s weight in kilograms divided by the square of height in meters.

### Statistical analyses

All the analyses were carried out in R version 1.2.5042. We used correlation and linear regression analysis to study the relationships between variables, and partial correlation network analysis to visualize them [30–32]. Results were adjusted for age and BMI, due to their association with health and life satisfaction [33, 34].

Bivariate correlation analysis was first used to check the zero order correlations between all the variables. Then, four different models were tested using both regression and network analysis methods. In linear regression analyses, life satisfaction was the dependent variable. In the first model, grip strength and OLST time were separately tested as independent variables. The second model included both grip strength and OLST time. In the third model, age and BMI were also added. The final (fourth) model included all the study variables (i.e., grip strength, OLST time, age, BMI, self-rated health, and self-rated mobility). The relationship of baseline measures to follow-up life satisfaction was also studied using multiple linear regression.

The network analysis (packages bootnet, v1.4.3 and qgraph, v1.6.5) was carried out in the same manner. In the case of a large sample size and low data dimension (number of variables), unregularized networks are used [35]. The partial correlation network is created by inverting the variance covariance matrix [36]. We tested multiple available algorithms and found that ggmModSelect provided the best sensitivity and specificity combination for our dataset and sample size [37]. Centrality indices were used to identify the important nodes in the network. In addition, a case-drop bootstrap was performed to test the stability of centrality indices at various sample sizes. The accuracy of the edge weights was assessed by testing the 95% CI around the edges using the non-parametric bootstrap method. The selection process and the structure of the network analysis are presented as supplementary material to this paper.

## Results

Baseline characteristics of the study population are presented in Table 1. The mean age of the participants in our study was 67.4 years. The mean life satisfaction score was 8.1 at baseline and 8.5 at follow-up. A great majority of the women (1413/1484 = 95%) reported moderate (*n* = 770), good (*n* = 535), or very good (*n* = 108) self-rated health, and 92% (1362/1485) were either fully capable of movement (*n* = 839) or able to walk but unable to run (*n* = 523).

**Table 1:** Baseline Characteristics of The Study Population (*n* = 1485)

	Mean (SD)	Range
Continuous variables		
Age (years)	67.4 (2.9)	62.6–72.8
Body mass index	28.6 (5.1)	16.6–61.7
Grip strength (kgF)	25 (5.8)	2.5–42
One-legged stance test (seconds)	18.2 (11.4)	0–30
Life satisfaction score^a^	8.1 (2.61)	4–20
Follow-up life satisfaction score^a^	8.5 (2.67)	4–20
Categorical variables		
Self-rated health^b^		1–5
Self-rated mobility^c^		1–6

^a^ Lower scores represent higher life satisfaction (range: 4-20)

^b^Subjects in each category: 1. poor (*n* = 2), 2. not good (*n* = 70), 3. moderate (*n* = 770), 4. good (*n* = 535), 5. very good (*n* = 108)

^c^Subjects in each category: 1. incapable of movement (*n* = 2), 2. can only move indoors (*n* = 7), 3. can walk 100 m at most (*n* = 24), 4. can walk 1 km at most (*n* = 90), 5. can move, unable to run (*n* = 523), 6. fully capable of movement (*n* = 839)

Zero order correlations between the variables are shown in Table 2. All the study variables were significantly correlated with baseline and follow-up life satisfaction, except BMI, which only correlated at follow-up. On both occasions, self-rated health and self-rated mobility were the two strongest correlates of life satisfaction, but their mutual correlation was still higher.

**Table 2:** Pearson’s correlation coefficients between all variables, where A) includes baseline life satisfaction (*n* = 1485) and B) includes follow-up life satisfaction (*n* = 1159)

A)						
	LS(BL)	GRIP	OLS	AGE	BMI	SRH
Life satisfaction (BL)						
Grip strength	0.12***					
One-legged stance	0.17***	0.26***				
Age	− 0.12***	− 0.23***	− 0.27***			
BMI	−0.05	0.03	− 0.33***	0.02		
Self-rated health	0.39***	0.17***	0.28***	−0.11***	−0.22***	
Self-rated mobility	0.31***	0.20***	0.37***	−0.18***	−0.28***	0.5***
B)						
	LS(FU)	GRIP	OLS	AGE	BMI	SRH
Life satisfaction (FU)						
Grip strength	0.09**					
One-legged stance	0.17***	0.23***				
Age	−0.15***	−0.22***	−0.27***			
BMI	−0.07*	0.02	−0.35***	0.03		
Self-rated health	0.27***	0.14***	0.26***	−0.11***	−0.24***	
Self-rated mobility	0.26***	0.14***	0.34***	−0.15***	−0.31***	0.49***

*LS* Life satisfaction, *OLS* One-legged stance test, *SRH* Self-rated health, *SRM* Self-rated mobility, *BL* Baseline, *FU* Follow-up

* *p* < 0.05, ** *p* < 0.01, *** *p* < 0.001

At baseline, both grip strength (β = − 0.051, *p* < 0.001) and OLST time (β = − 0.037, *p* < 0.001) had a significant linear relationship with life satisfaction, whereas in the multiple linear regression analysis, this was true for self-rated health (β = − 4.376, *p* < 0.001), age (β = − 0.046, *p* = 0.043), and BMI (β = − 0.035, *p =* 0.009) (Table 3). In the multiple regression analysis with follow-up life satisfaction as the dependent variable (Table 4), only self-rated health (β = − 5.05, *p =* 0.003) and age (β = − 0.088, *p =* 0.001) had a significant relationship with it.

**Table 3:** Univariate (Model 1) and Multiple (Models 2, 3, and 4) Linear Regression Analyses with Baseline Life Satisfaction Score as the Dependent Variable (*n* = 1485)

	β	Std. error	Adj. r squared	*p*-value
Model 1 (univariate)				
Grip strength	−0.051	0.011	0.013	< 0.001
One-legged stance test	−0.037	0.006	0.027	< 0.001
Model 2			0.032	
Grip strength	−0.035	0.012		0.004
One-legged stance test	−0.034	0.006		< 0.001
Model 3			0.036	
Grip strength	−0.029	0.012		0.014
One-legged stance test	−0.030	0.007		< 0.001
Age	0.065	0.024		0.007
Body mass index	0.004	0.014		0.783
Model 4			0.183	
Grip strength	−0.004	0.011		0.733
One-legged stance test	−0.007	0.006		0.27
Age	0.046	0.022		0.043
Body mass index	−0.035	0.013		0.009
Self-rated health	−4.376	1.272		< 0.001
Self-rated mobility	−1.081	1.237		0.382

**Table 4:** Multiple Linear Regression Model with Follow-up Life Satisfaction Score (10 Years after Baseline) as the Dependent Variable (*n* = 1159)

	β	Std. error	Adj. r squared	*p*-value
Model			0.091	
Grip strength	−0.004	0.014		0.728
One-legged stance test	−0.0133	0.007		0.085
Age	0.088	0.027		0.001
Body mass index	−0.017	0.016		0.294
Self-rated health	−5.05	1.702		0.003
Self-rated mobility	−0.242	0.868		0.780

**Fig. 2:**
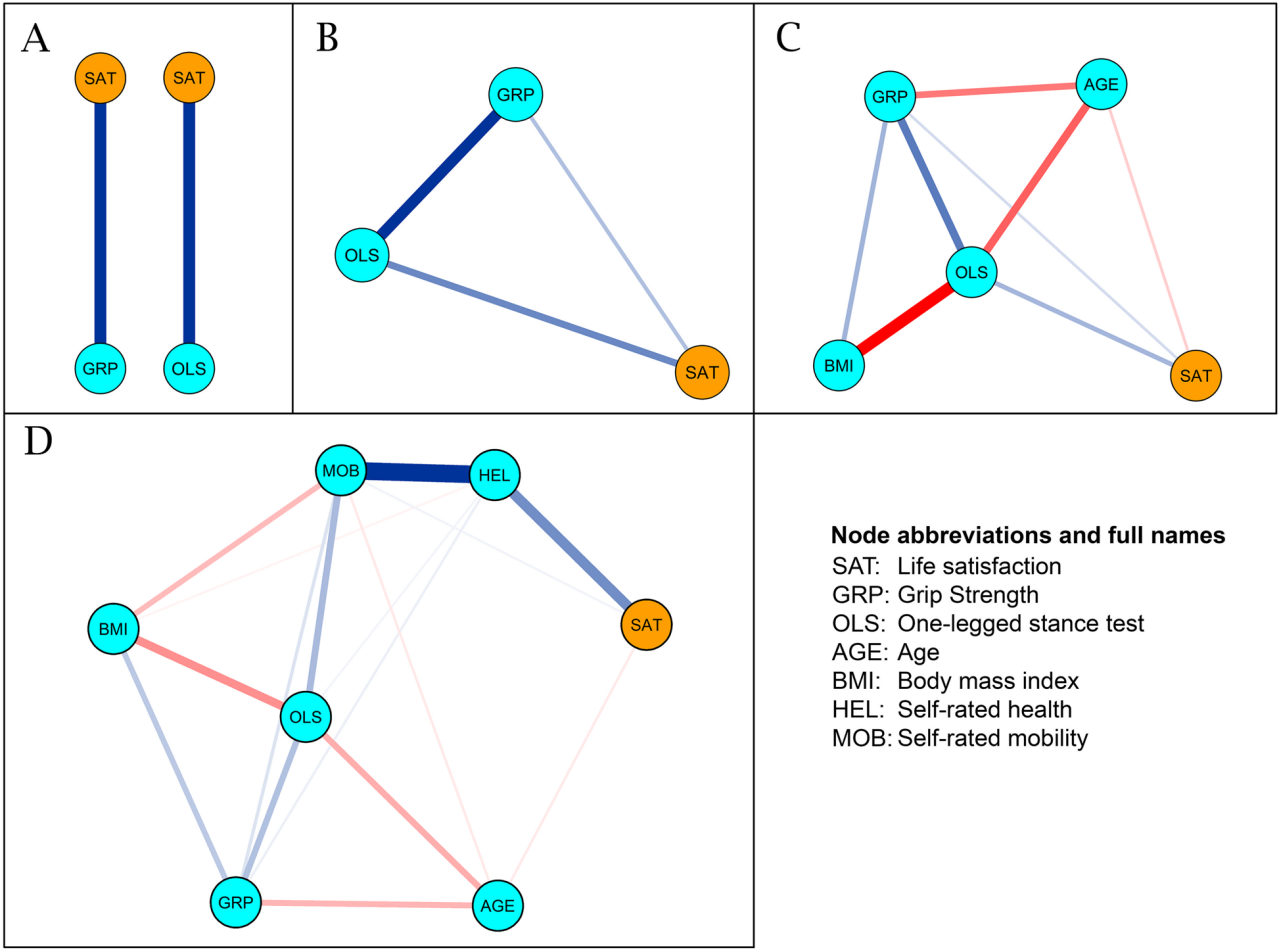
Partial correlation network analyses demonstrating relationships between grip strength, one-legged stance test, BMI, age, self-rated health, self-rated mobility, and life satisfaction. The partial correlation network consists of nodes representing the variables and the edges connecting them, which represent the partial correlation coefficient between the two nodes. Edges with positive correlation coefficient are colored blue, whereas edges with negative correlation coefficient are colored red. The thickness and color of the edges indicate the strength of the correlation coefficient between the two nodes

The partial correlation network analyses demonstrating cross-sectional relationships between all the variables are shown in Fig. 2. In the first model (A), both grip strength (*r* = 0.11) and OLST time (*r* = 0.17) were separately correlated with life satisfaction. In the second model (B), which included both grip strength (*r* = 0.08) and OLST time (*r* = 0.14) together, both of them correlated with life satisfaction, but more strongly with each other (*r* = 0.24*)*. In the third model (C), after adding BMI and age as well, OLST time (*r* = 0.12) and grip strength (*r* = 0.06) still significantly correlated with life satisfaction. After the inclusion of self-rated health and self-rated mobility in the model (D), the correlation of OLST time and grip strength with life satisfaction was lost. In the model (D), OLST time correlated positively with self-rated mobility (*r* = 0.20) and grip strength (*r* = 0.19), but weakly with self-rated health (*r* = .03), and negatively with age (*r* = − 0.18) and BMI (*r* = − 0.25). Grip strength correlated positively with BMI (*r* = 0.16), self-rated mobility (*r* = 0.08), and self-rated health (r = 0.05), and negatively with age (*r* = − 0.15). Self-rated health had a strong correlation with life satisfaction (*r* = 0.31), but the strongest was with self-rated mobility (*r* = 0.57). BMI and age had a negative correlation with mobility (*r* = − 0.16 and − 0.05, respectively).

Self-rated mobility, self-rated health, and OLST time were the most central nodes in the network (Supplementary Figs. 1 and 2). The results for the centrality indices were stable (Supplementary Fig. 3). Even after a drop of up to 65–75% in sample size, the centrality indices retained a correlation of 0.7 with the original results. The edge weight accuracy results show 8 edges that were most reliable, with narrow bootstrapped confidence intervals (Supplementary Fig. 4). The two strongest edges in the network (1. self-rated health <− > self-rated mobility, 2. life satisfaction <− > self-rated health) were significantly stronger than every other edge in the network (Supplementary Fig. 5).

## Discussion

Physical tests such as grip strength and OLST time had no direct association with life satisfaction but were associated with self-rated mobility in older Finnish women when subjective health was also in the model. As expected, the strongest correlate of present and long-term life satisfaction was self-rated health. Thus, women who have lower subjective health are more likely to be more dissatisfied in current and later life.

To our knowledge, there are few studies investigating the association between physical capacity and life satisfaction. A positive association in men but not in women has been reported between grip strength and SWB, measured with the 17-item revised “Philadelphia Geriatric Center (PGC) Morale Scale” [20]. In another previous study, grip strength was positively associated with life satisfaction, but self-rated health and other covariates highly correlated with life satisfaction were not included the model [33]. We could not find any previous studies on association of balance capacity tests with life satisfaction, but a strength and balance training program has demonstrated an improvement in life satisfaction [38].

In our study, both in regression and network univariate analyses, the inclusion of two subjective measures (health and mobility) in the model removed the significant association between grip strength and life satisfaction, as well as between OLST time and life satisfaction. This is understandable, since life satisfaction is also a subjective measure, and correlates strongly with other subjective measures of health and wellbeing. However, it does not rule out the indirect effects of physical capacity on life satisfaction. It has been suggested that the effect of frailty (which has grip strength and walking speed as its components) on life satisfaction is mediated through changes in self-rated health [39].

Previous studies have investigated health and self-rated health in respect of life satisfaction. One study found that self-rated health explained around 14% of variance in the LSI-Z (i.e., a 13-item life satisfaction index) among frail older adults [40]. A meta-analysis of health and SWB concluded that health (subjective and objective) explained 4-16% of the variance in SWB in the general population [41]. In our results, too, higher self-rated health, meaning higher life satisfaction currently and in the 10-year follow-up, is as expected.

There was no independent association between self-rated mobility and life satisfaction in multiple regression analysis. In the network analyses, it was present but weak. The reason for the lack of association could be the homogeneous mobility status in our cohort, with 92% of the women capable of normal movement and 98% being able to walk up to 1 km. Mobility could have a stronger effect on life satisfaction at ages when mobility limitations start to occur, but this warrants future longitudinal studies [42]. Previously, self-rated mobility has demonstrated association with life satisfaction, when not adjusted for self-rated health [41, 43].

In general, the results from both the regression and network analyses were congruent. The strongest relationships were similar, but some differences can be expected with different techniques. Whether or not a relationship is drawn between two nodes depends on the thresholds set by the network analysis method, which can be adjusted to have either conservative or relaxed estimates. However, the network analysis provides a visual demonstration of how the variables interact, which makes it a viable option for clinical research.

Self-rated health, self-rated mobility, and life satisfaction are self-reports and subject to biases such as recall bias and common method variance, meaning “variance that is attributable to the measurement method rather than to the constructs the measures represent” [44, 45]. The results for analyses with follow-up life satisfaction are less prone to it because the dependent and independent variables were measured at different points in time. Further, there is bias created by the instrument used. A variety of instruments for life satisfaction are available, and their differences should be noted. Domain-specific satisfactions do not measure global life satisfaction. Indeed, it is always crucial to understand what has been assessed by a scale. However, it has been suggested that people may answer in a consistent manner on *alternative* items and scales, which may lead to moderate to high correlation between different life satisfaction scales [46].

In the present study, we used the 4-item life satisfaction scale that was developed in 1973. It has a solid research base with studies on its stability, cross-sectional associations for relevant psychometric scales, and longitudinal analysis of its outcomes with different study populations [6–14, 47]. The three items of the scale inquired about happiness, interest, and ease in life. Only the loneliness scale was asking about feelings of loneliness, not loneliness *in life*. Since happiness and life satisfaction are indicators of subjective wellbeing, one could also call the 4-item life satisfaction scale a subjective wellbeing measure. However, since it has been used in the same form as in the present study since the 1970s, we did not change it. In general, it is important to acknowledge what is being assessed before comparing studies utilizing different SWB or life satisfaction instruments.

Both the strengths and limitations of our study and its self-reported measures should be acknowledged. The results of our study are limited to post-menopausal Finnish women. There are gender and cross-cultural differences in life satisfaction, and therefore similar studies need to be carried out for men and in different settings. The focus of our study was to compare objective and subjective measures of health in respect to life satisfaction. The 4-item life satisfaction has a very high response rate (≥95%) to all items and a linear trend in the response alternatives with respect to various health adversities [48]. Removing study subjects with missing data and with a “cannot say” response from the analysis did not change the results (Supplementary Tables 1 and 2). Self-reported measures can provide important knowledge on perceived health and wellbeing, but also on objective health.

## Conclusion

Our results highlight that self-reported measures can provide important knowledge not only about perceived health and wellbeing, but also about objective health. Self-reports can enable early detection and intervention in health hazards in old age, when physical capacity and mobility are crucial in maintaining independence and social contacts. In older Finnish women, the self-reports of health, mobility, and life satisfaction were closely intertwined. Objective physical capacity, even if associated with current and long-term life satisfaction, lost this association when self-rated health and self-rated mobility were introduced in the same model, but it was still associated with self-rated mobility. Thus, the impact of objective physical capacity measures on subjective wellbeing and their indirect associations, as well as differences in this association across ages, cultures, and genders, warrant further longitudinal studies among aging people.

## Supplementary Information

**Additional file 1.**

## Data Availability

The datasets used and/or analyzed during the current study are available from the corresponding author on reasonable request.
