# Supplementary material for: Physical capacity, subjective health, and life satisfaction in older women: a 10-year follow-up study
Source: BMC Geriatr. 2021 Nov 23;21:658. doi: 10.1186/s12877-021-02605-z (PMC8609741; doi:10.1186/s12877-021-02605-z)
Supplement: Supplementary file 1 — Additional file 1. [file 12877_2021_2605_MOESM1_ESM.docx]

**Supplementary material**

**Supplementary Figure 1. Comparison of correlation, sensitivity, and specificity results for different partial correlation network analysis methods at various sample sizes.**

**
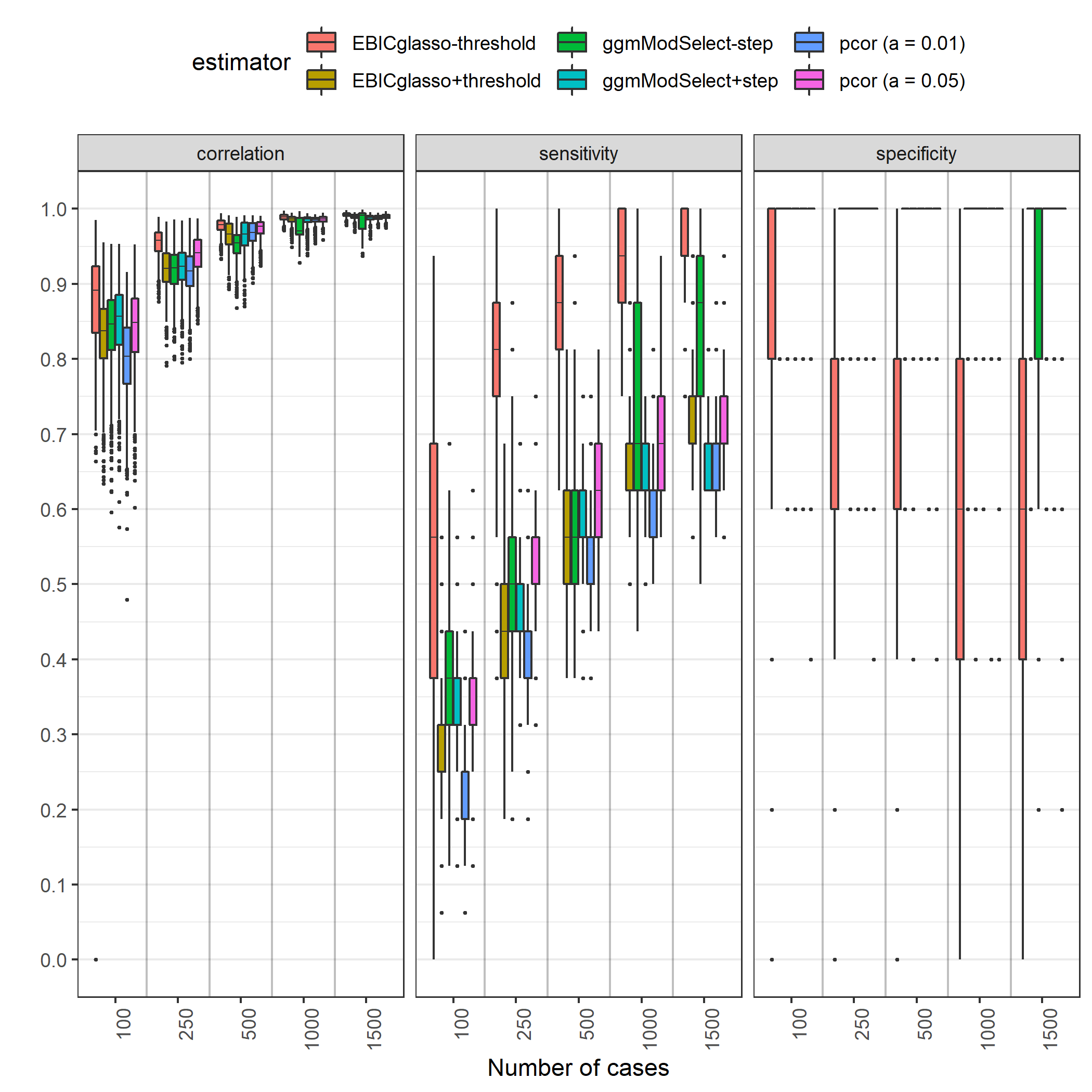
**

EBICglasso, ggmModSelect, and simple partial correlation were tested in a simulation. Correlation between results, sensitivity, and specificity were tested at multiple sample sizes (100, 250, 500, 100, 1500). Simulations were repeated 1500 times. The ggmModSelect method (without steps) displayed a good balance between sensitivity and specificity.

**Supplementary Figure 2. Betweenness, strength, and closeness centrality indices for the final network model (Figure 2D).**


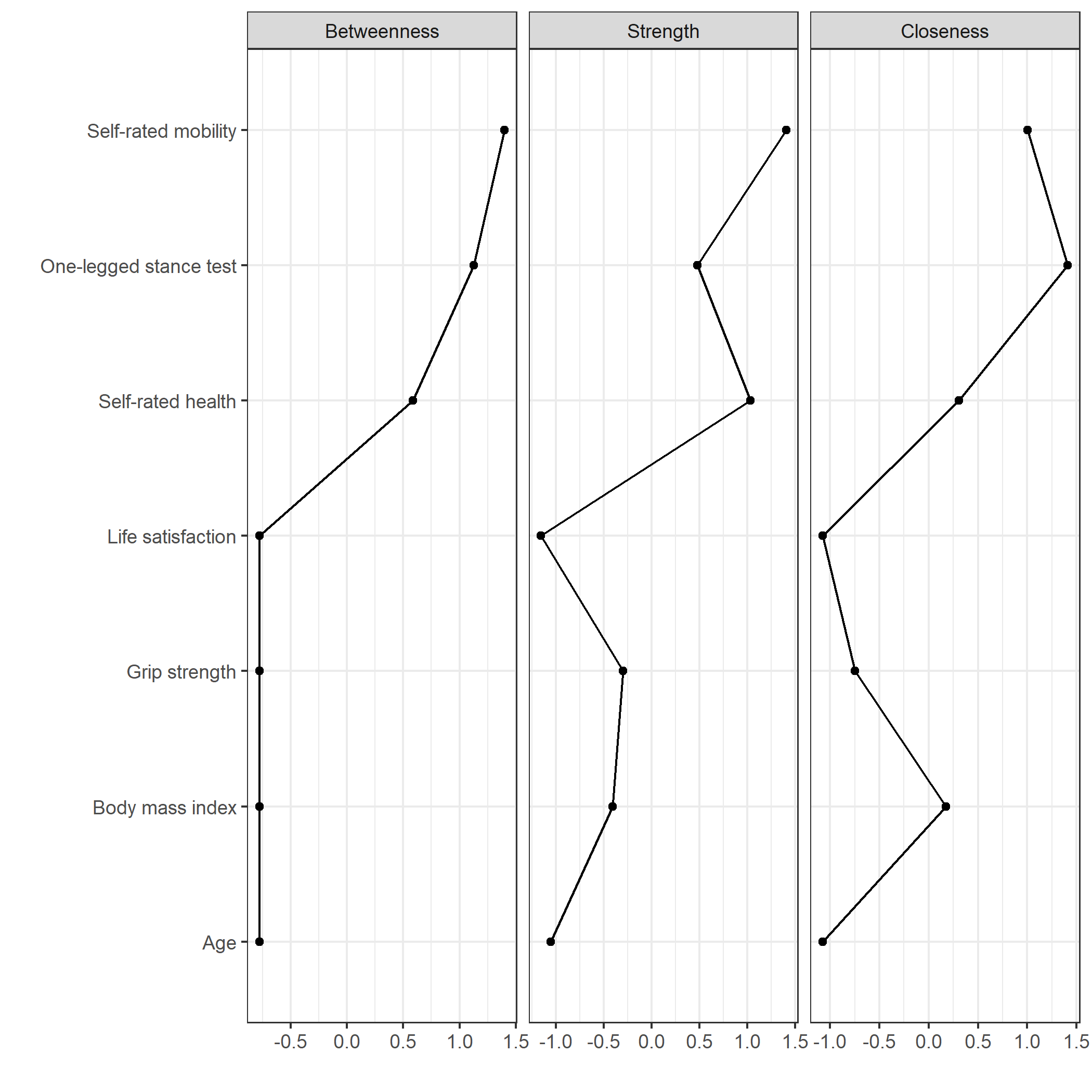


Strength centrality identifies nodes with strongest connections to their neighboring nodes. Closeness centrality identifies nodes that have a strong connection to all nodes. Betweenness centrality is based on the number of shortest inter-node pathways that pass through a certain node. Self-rated mobility, self-rated health and one-legged stance test score were the most central nodes in the network.

**Supplementary Figure 3. Case-drop bootstrap analysis results for stability of the centrality indices.**


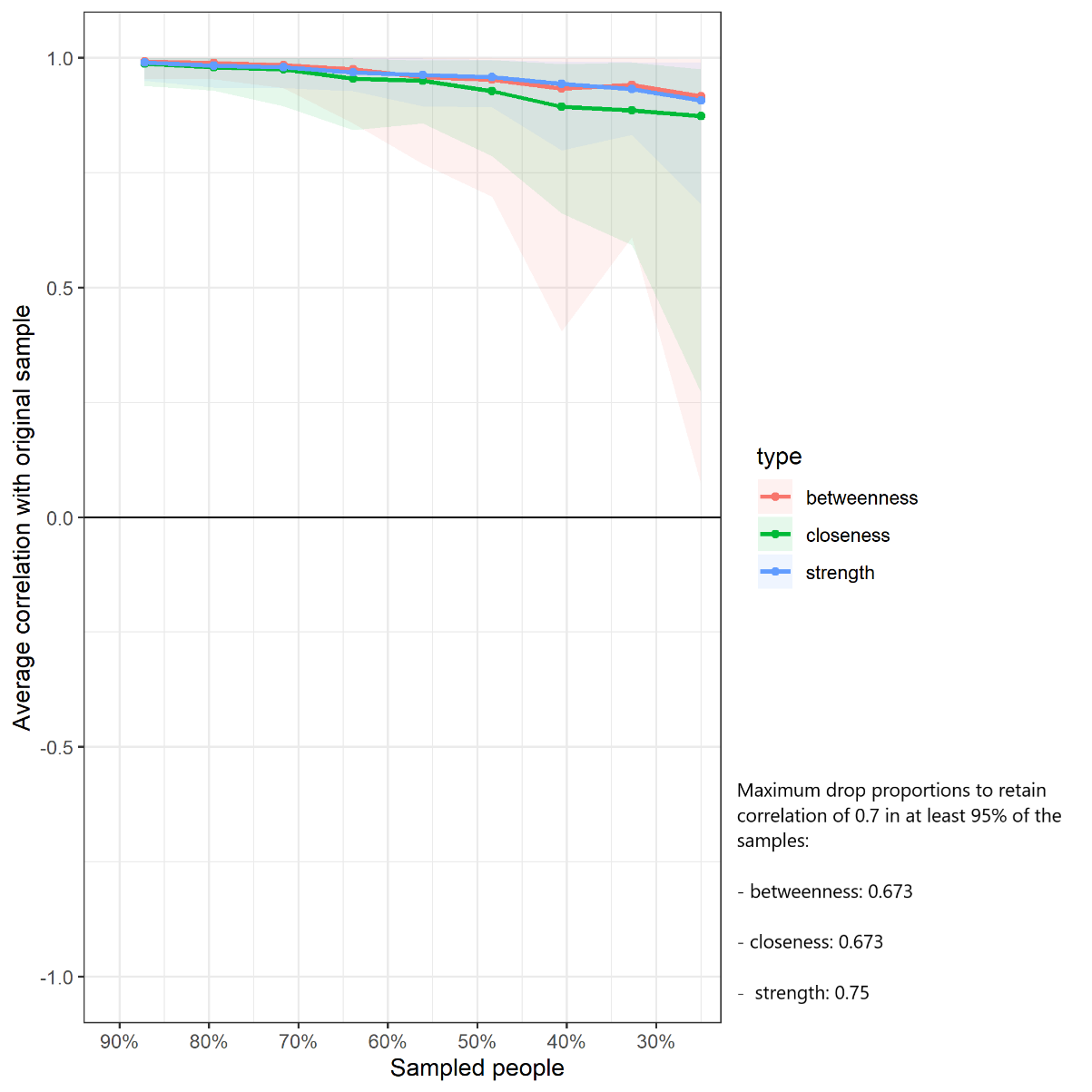


Stability of the centrality indices for the estimated network. Centrality indices were reliable, because in 95% of bootstrap samples, even after dropping up to 65–75% of cases, the results for centrality indices retained a correlation of 0.7 with the original results.

**Supplementary Figure 4: 95% CI around the edge weights (correlation coefficients) between the study variables.**

**
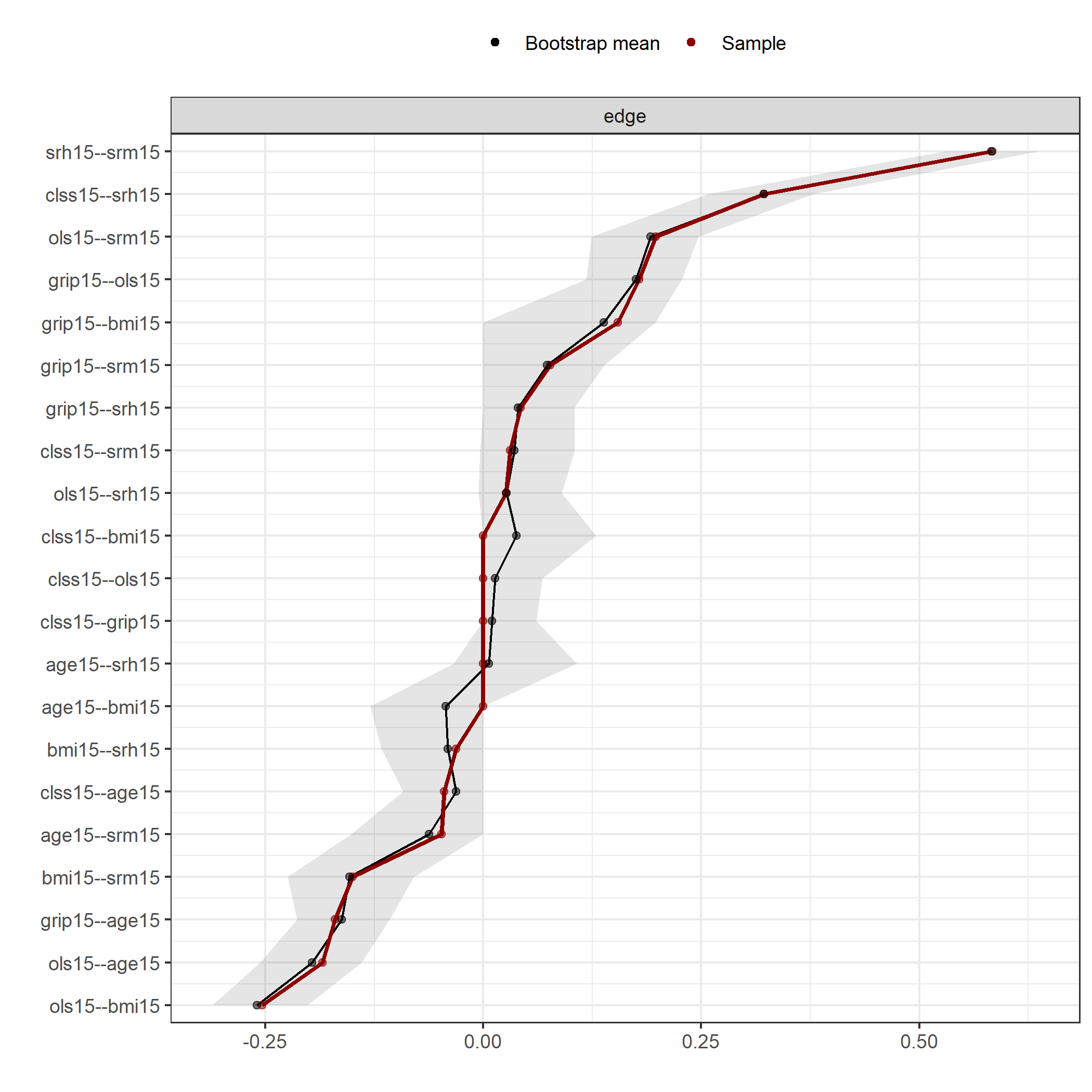
**

Edge weights from our results (red) are compared with the mean from bootstrap results (black). The gray area indicates the bootstrapped confidence interval for each edge. Bootstrapped CI for edges (1) srh – srm, (2) srh – clss, (3) ols – srm, (4) grip – ols, (5) ols – bmi, (6) ols – age, (7) grip – age, and (8) bmi – srm are the most reliable, as the bootstrapped CI around these edges is narrow. (srh = self-rated health, srm = self-rated mobility, clss = life satisfaction score, ols = one-legged stance test, grip = grip strength, bmi = body mass index)

**Supplementary Figure 5: Statistical difference tests for edge weights between the variables studied.**


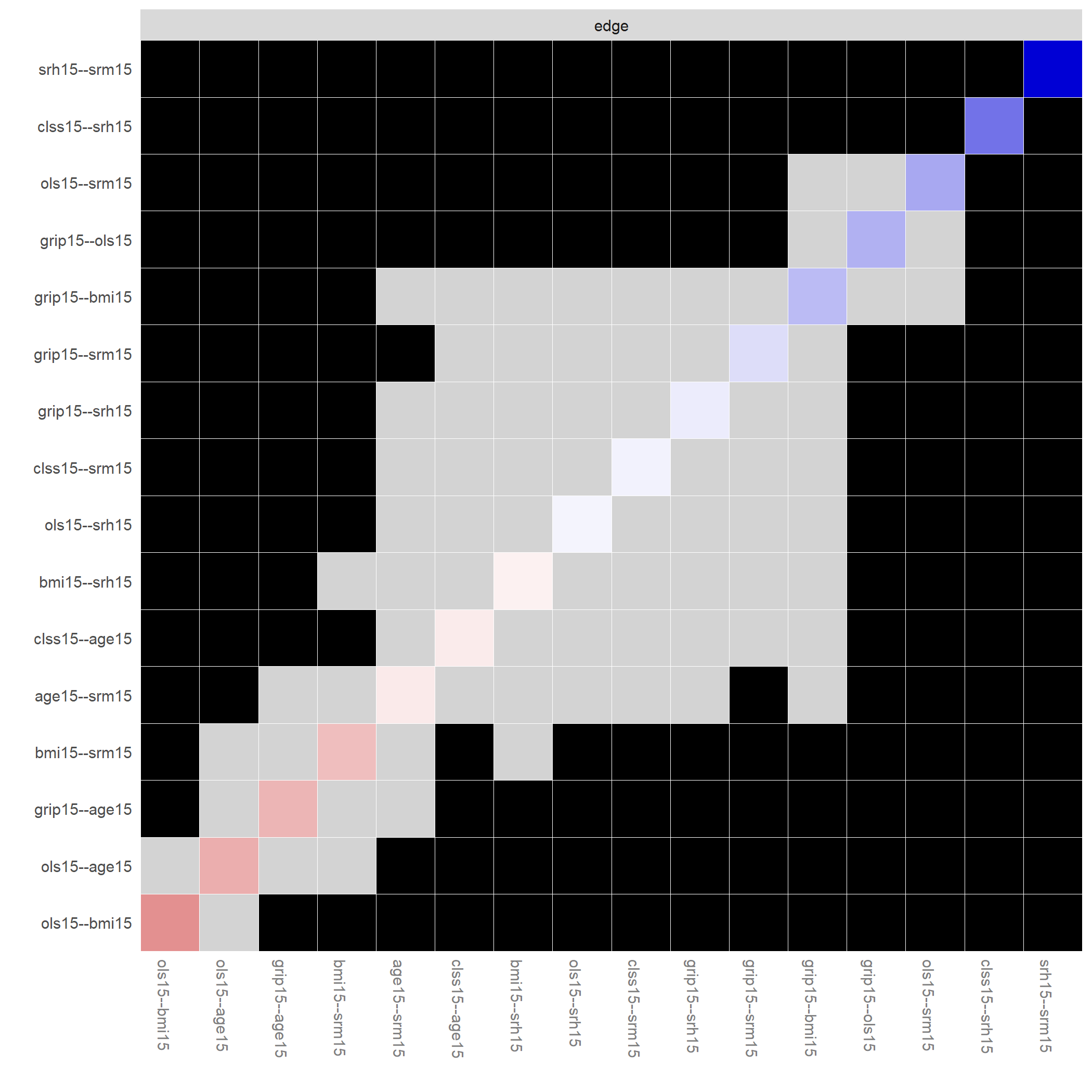


The figure compares bootstrapped confidence intervals of edge. If they did not overlap in more than 95% of instances, they were considered significantly different. Black squares indicate significantly different edges, and gray squares indicate that the edge weights were similar. For instance, we can see that the correlation between self-rated health and self-rated mobility was significantly stronger than the correlation between self-rated health and the life satisfaction score. (srh = self-rated health, srm = self-rated mobility, clss = life satisfaction score, ols = one-legged stance test, grip = grip strength, bmi = body mass index)

**Supplementary Tables**

After omitting the answers of “cannot say” and the missing cases, the data was reanalyzed. The results are given below (Supplementary Tables 1 and 2) where there is a side-by-side comparison of these with the previous results. The sample size decreased substantially, 75.2% of subjects at baseline (1122 out of 1485) and 76.8% of subjects in the follow-up (890 out of 1159) gave answers for all life satisfaction components. Even after the decrease in sample size, the results stayed the same. There was a change in β constant values for SRH and SRM because we had to remove category 1, since there were no subjects remaining in that category. But that did not affect the significance of the results.

| **Supplementary Table 1.** Multiple Linear Regression Model with Baseline Life Satisfaction Score as the Dependent Variable (*n* = 1485) -> After removal of missing cases and answers of “cannot say”, *n* = 1122 | | | | | | |
| --- | --- | --- | --- | --- | --- | --- |
|  | β | β2 | Std. error | Std. error2 | *p*-value | *p*-value2 |
| Model | n= | n= | n= | n= | n= | n= |
| Grip strength | -0.004 | 0.005 | 0.011 | 0.006 | 0.733 | 0.49 |
| One-legged stance test | -0.007 | 0.001 | 0.006 | 0.003 | 0.27 | 0.836 |
| Age | 0.046 | 0.029 | 0.022 | 0.013 | 0.043 | 0.029 |
| Body mass index | -0.035 | -0.021 | 0.013 | 0.008 | 0.009 | 0.011 |
| Self-rated health | -4.376 | -2.01 | 1.272 | 0.186 | <0.001 | <0.001 |
| Self-rated mobility | -1.081 | -0.356 | 1.237 | 0.511 | 0.382 | 0.484 |

| **Supplementary Table 2.** Multiple Linear Regression Model with Follow-up Life Satisfaction Score (10 Years After Baseline) as the Dependent Variable (*n* = 1159). -> After removal of missing cases and answers of “cannot say, *n* = 890 | | | | | | |  |
| --- | --- | --- | --- | --- | --- | --- | --- |
|  | β | β2 | Std. error | Std. error2 | *p*-value | *p*-value2 | |
| Model |  |  |  |  |  |  | |
| Grip strength | -0.004 | -0.004 | 0.014 | 2.45 | 0.728 | 0.208 | |
| One-legged stance test | -0.0133 | -0.008 | 0.007 | 0.008 | 0.085 | 0.375 | |
| Age | 0.088 | 0.085 | 0.027 | 0.031 | 0.001 | 0.007 | |
| Body mass index | -0.017 | -0.011 | 0.016 | 0.018 | 0.294 | 0.545 | |
| Self-rated health | -5.05 | -2.26 | 1.702 | 0.443 | 0.003 | <0.001 | |
| Self-rated mobility | -0.242 | 1.301 | 0.868 | 0.656 | 0.780 | 0.446 | |
